# Efficient adsorptive removal of paracetamol and thiazolyl blue from polluted water onto biosynthesized copper oxide nanoparticles

**DOI:** 10.1038/s41598-023-28122-0

**Published:** 2023-01-17

**Authors:** Kovo G. Akpomie, Jeanet Conradie

**Affiliations:** 1 Physical Chemistry Unit, Department of Chemistry, University of the Free State, Bloemfontein, South Africa; 2 Industrial/Physical Chemistry Unit, Department of Pure and Industrial Chemistry, University of Nigeria, Nsukka, Nigeria

**Keywords:** Environmental sciences, Chemistry, Engineering, Materials science

## Abstract

Copper oxide nanoparticles (CuONPs) have received tremendous attention as efficient adsorbents owing to their low cost, desirable surface area, abundant active sites, potent textural characteristics and high adsorption capacities. However, CuONPs have not been employed to decontaminate water laden with increasing environmental contaminants such as thiazolyl blue and paracetamol. Herein, the adsorption of thiazolyl blue and paracetamol onto green synthesized CuONPs prepared from the aqueous leaf extract of *Platanus occidentalis* was studied. The BET, SEM, FTIR, XRD, EDX and pH point of zero charge showed the successful synthesis of CuONPs having desirable surface properties with a surface area of 58.76 m^2^/g and an average size of 82.13 nm. The maximum monolayer adsorption capacities of 72.46 mg/g and 64.52 mg/g were obtained for thiazolyl blue and paracetamol, respectively. The Freundlich, pseudo-second-order and intraparticle diffusion models were well fitted to the adsorption of both pollutants. The pH studies suggested the predominance of electrostatic and weaker intermolecular interactions in the adsorption of the thiazolyl blue and paracetamol, respectively. Spontaneous, physical, endothermic and random adsorption of the pollutants on CuONPs was obtained from the thermodynamic consideration. The biosynthesized CuONPs were found to be highly reusable and efficient for the adsorption of thiazolyl blue and paracetamol from water.

## Introduction

In order for all living things to be healthy and happy, there is a need for clean water. In most developing and wealthy countries, the lack of clean water has become a critical issue^1^. This is attributed to rapid technological advancement, industrial effluent pollution, the rapid growth of the human population, and erratic rainfall which degrades water quality^2–4^. Regular use of contemporary amenities and goods such as gasoline for cars, plastics, textile apparel, food products, and medications, to name a few has led to enormous waste product generation and contamination of previously unpolluted water basins^5^. The use of contaminated water has had serious negative impacts on vegetation, people and other living things^6^. In developing nations, the local population's most dependable source of clean water, which is groundwater, is being rapidly polluted by industrial discharges of both organic and inorganic effluent^7^.

Dyes and pharmaceutical pollutants are one of the most common water contaminants^8,9^. The largest user of dyes, with annual global use of around 700,000 tons, is the textile sector^10^. As a result, a sizeable number of dyes are discharged into the environment from wastewaters used in the textile industry^11^. The dyes discharged can negatively impact the environment and as well as human health. Due to their high stability, which encourages resistance to biological and photo-degradation, dyes are challenging to remove from water^12^. Additionally, they lessen the amount of light that enters water bodies, which affects how aquatic plants use light for photosynthesis. Additionally, the kidney, liver, brain, skin, central nervous system, and reproductive system may be harmed by their breakdown products, which can also be mutagenic, and carcinogenic^13^. They can also cause health problems such as nausea, vomiting, eye burn and breathing difficulties^11^. Most studies have focused on the removal of dyes such as malachite green, rhodamine B, crystal violet, methyl orange, eriochrome black t, Congo red, and methylene blue from water with limited research on other potentially harmful dyes like thiazolyl blue, which should be considered^14^. Moreover, pharmaceutical contaminants are also harmful to the environment and human health^15^. In particular, acetaminophen, usually referred to as paracetamol (N-(4-hydroxyphenyl)acetamide) is one of the most used analgesics and antipyretics drugs in the world. It has been found globally in surface waters, effluent, and drinking water because of its great stability, solubility, and hydrophilicity^16^. This drug's overuse results in several physiological issues. A portion of the medicine that is not metabolized is excreted through urine after administration. As a result, this pollutant may be present in varying amounts in the home or hospital effluents. Because the medication can build up in adipose tissue to quantities capable of producing biological activity, the presence of bioactive compounds in water for human consumption creates long-term toxicological hazards^17^. Therefore, in order to preserve environmental water quality, it is necessary to treat wastewaters contaminated with thiazolyl blue and paracetamol.

Generally, techniques such as forward and reverse osmosis, advanced oxidation, electrochemical degradation, ultrasonic degradation, UV-degradation, ion exchange, solvent extraction, filtration, ozonation, coagulation-flocculation, photo-Fenton process, biological treatment and adsorption have been used for the treatment of wastewaters polluted with dyes and pharmaceuticals^16,18,19^. One of the main drawbacks of these technologies is the high expense, complexity and requirement for ongoing monitoring^20^. The adsorption method is one of the most preferred because of how easy, flexible, inexpensive, and effective it is^21,22^. Therefore, several studies have been conducted on the adsorption of dyes and pharmaceuticals from water as a cost-efficient procedure^23–26^.

In recent years, the adsorption of pollutants from wastewater onto nano-adsorbents such as copper oxide nanoparticles (CuONPs) has received tremendous attention owing to the monoclinic structure, narrow band gap, low cost, high surface area, abundant active sites, cytotoxicity, catalytic activity, desirable textural properties, antimicrobial activities and efficient adsorption capacity^27–31^. In a previous investigation, the adsorption of basic violet and basic red onto CuONPs exhibited a maximum adsorption capacity of 16.86 mg/g and 27.24 mg/g, respectively^32^. Another report showed that biosynthesized CuONPs had a maximum monolayer adsorption capacity of 36.52 mg/g for methylene blue^33^. Ahmad et al.^34^ obtained a high CuONPs adsorption capacity of 105 mg/g for ciprofloxacin. In other reports, CuONPs exhibited high adsorption of 3152 mg/g for fluoride^35^, 144.4 mg/g for congo^36^ and 825.2 mg/g for mercury ions^37^. However, the high adsorption potential of CuONPs is yet to be exploited in the adsorption of thiazolyl blue and paracetamol from water.

There are several chemical and physical procedures that can be used to make CuONPs, including sol–gel, polyethylene-glycol-dependent, hydrothermal, thermal decomposition, microwave irradiation, precipitation, sonochemical and electrochemical processes^27,28^. These techniques are time-consuming, expensive, and complex. They also need tremendous pressure and temperature, and the stabilizers and chemicals they utilize are toxic^27^. The biological or green synthesis of nanoparticles using plant extracts, yeast, algae, fungi or bacteria is preferred because of the biocompatibility, higher yields, simplicity, low cost, rapidness, sustainability, environmental friendliness and non-toxicity^28,30,38,39^. Therefore, this study was focused on the green synthesis of CuONPs via the aqueous leave extract of *Platanus occidentalis* for the adsorption of thiazolyl blue and paracetamol from solution. Every winter, the *Platanus occidentalis* tree sheds its leaves, creating a huge number of debris that is of no real use^14^. Therefore, valorizing the leaf waste in the synthesis of nanoparticles has advantages for reducing waste and protecting the environment. The biosynthesized CuONPs were characterized to determine the surface properties which could influence adsorption. Batch adsorption was used to examine the influence of temperature, time, concentration and pH on the pollutant’s removal. Moreover, the adsorption behavior of the biosynthesized CuONPs was analyzed by suitable model equations and the regeneration and reuse were investigated.

## Materials and methods

### Materials and chemicals

All methods were performed in accordance with the relevant guidelines and regulations. The fallen dry *Platanus occidentalis* leaf wastes were collected from the premises of the University of the Free State, Bloemfontein Campus, South Africa. The leaves were identified by Dr. Andri Van Aardt of the Department of Plant Sciences of the University and declared not endangered^14^. The leaves fall of the tree during winter and constitute waste to the environment which are swept by cleaners to keep the environment tidy. The chemicals such as sodium hydroxide (NaOH), hydrochloric acid (HCl), thiazolyl blue (C_18_H_16_BrN_5_S) and copper sulphate (CuSO_4_) were obtained from Sigma Aldrich, South Africa. The paracetamol (C_8_H_9_NO_2_) was obtained from Juhel pharmaceutical company, Emene, Enugu State, Nigeria. All the chemicals were used as purchased without any purification.

### Preparation of the leaf extract

The *Platanus occidentalis* leaves were divided into smaller pieces and washed with tap water in order to increase the surface area and get rid of any surface impurities. Then, 20 g of the leaves were combined with 100 ml of distilled water in a beaker to make the plant leaf extracts. After that, the mixture was heated for 90 min at 70 °C while being regularly stirred. The filtered and cooled *Platanus occidentalis* leaf extract was kept at 4 °C in the refrigerator and utilized for synthesis within a week.

### Biosynthesis of CuONPs and characterization

The CuONPs were synthesized by the green approach using the method described by Rather and Sundarapandian^27^ but with some modifications. This was accomplished by combining the obtained leaf extract in a 1:9 ratio with a solution of 0.01 M anhydrous copper sulphate. For 30 min, the mixture was continually stirred at room temperature. Thereafter, 0.01 M NaOH solution was added dropwise until pH 8.0 was achieved with further stirring for 90 min to aid the precipitation of the CuONPs. The solution was allowed to settle for 24 h and the resulting colloidal solution was centrifuged at 10,000 rpm for 30 min to create a pellet, which was then purified by washing it three times with distilled water. For moisture removal, the purified nanoparticles were dried in an oven at 70 °C for 12 h. The dried product was then calcined at 250 °C in a muffle furnace and powdered to obtain the biosynthesized CuONPs. The as-synthesized nanoparticles were characterized by the pH drift technique to determine the pH point of zero charge as described previously^40^. The nanoparticles were also characterized by the X-ray diffractometer (XRD; Brucker D8-Discover model), Scanning electron microscopy (SEM; Joel JSM-7800F model), Fourier transform infrared spectroscopy (FTIR; Brucker model), Brunauer-Emmet-Teller (BET) surface area and porosity analyzer (Micromeritics ASAP 2020 model) and the Energy dispersive X-ray spectroscopy (EDX; Oxford X-max model).

### Batch adsorption

Using the batch adsorption method, the removal of the dye and pharmaceutical from the solution onto CuONPs was investigated. Here, a stock solution of each (thiazolyl blue and paracetamol) containing 100 mg/L was created by adding the required amounts of each substance to distilled water. Lower concentrations from the stock, ranging from 20 to 80 mg/L, were then generated by applying sequential dilutions. The pH of the solutions was changed from 2.0 to 10 using 0.1 M sodium hydroxide and hydrochloric acid solutions. The influence of sonication time (5–120 min), pH (2.0–10.0), adsorbate concentration (20–100 mg/L) and temperature (300–320 K) on adsorption onto the biosynthesized CuONPs was investigated. In the standard batch process, 20 mg of CuONPs were added to 10 mL of the prescribed solution at 300 K, pH 8.0 for thiazolyl blue and pH 7.0 for paracetamol. Before filtering, the contacting solution was sonicated for 120 min. The remaining concentrations of thiazolyl blue and paracetamol in the filtrate after centrifugation at 8000 rpm for 20 min were quantified using a UV-spectrophotometer (Shimadzu-1800 model) at the maximum wavelengths of 243 nm and 245 nm, respectively. To ascertain each factor's effect on adsorption, the researched factor of interest was altered while the other components were maintained at their optimum values. The equations used to estimate the adsorption capacity of CuONPs for the adsorbates and the percentage removal are presented in the supplementary document. For quality assurance and reproducibility, each experiment was carried out twice, with the average value computed. The error bars in the figures represent the standard deviation from the averages. Furthermore, the application of appropriate model equations as indicated in the supplementary document was used to perform the isotherm kinetics and thermodynamic modeling of the adsorption.

### Desorption and reuse

The desorption of thiazolyl blue and paracetamol from the loaded CuONPs was accomplished using 0.1 M HCl and 0.1 M NaOH as eluents, respectively, in order to regenerate the adsorbent. In this technique, 10 mL of the eluent was added to 20 mg of the adsorbate-loaded CuONPs for 10 min while being constantly stirred. The eluent containing the desorbed adsorbate was then decanted after it had settled for a while. The regenerated nanoparticles were washed with excess distilled water until neutral pH. The regenerated CuONPs were then reused for drug and dye adsorption after being dried for two hours at 100 °C in an oven. The adsorption procedure involved contacting the regenerated CuONPs with 10 mL of 100 mg/L adsorbate concentrations of thiazolyl blue at pH 8.0 and paracetamol at pH 7.0, at 300 K and 120 min of sonication time. The percentage removal of the adsorbate by the regenerated biosynthesized CuONPs was then calculated. The adsorption–desorption experiment was run five times to examine the ability of CuONPs to be reused in the adsorption process. Moreover, the adsorption–desorption experiments were conducted twice and the average values were calculated. The standard deviations from the averages were also represented by the error bars in the figures.

## Results and discussion

### Characterization of the copper oxide nanoparticles

**Figure 1:**
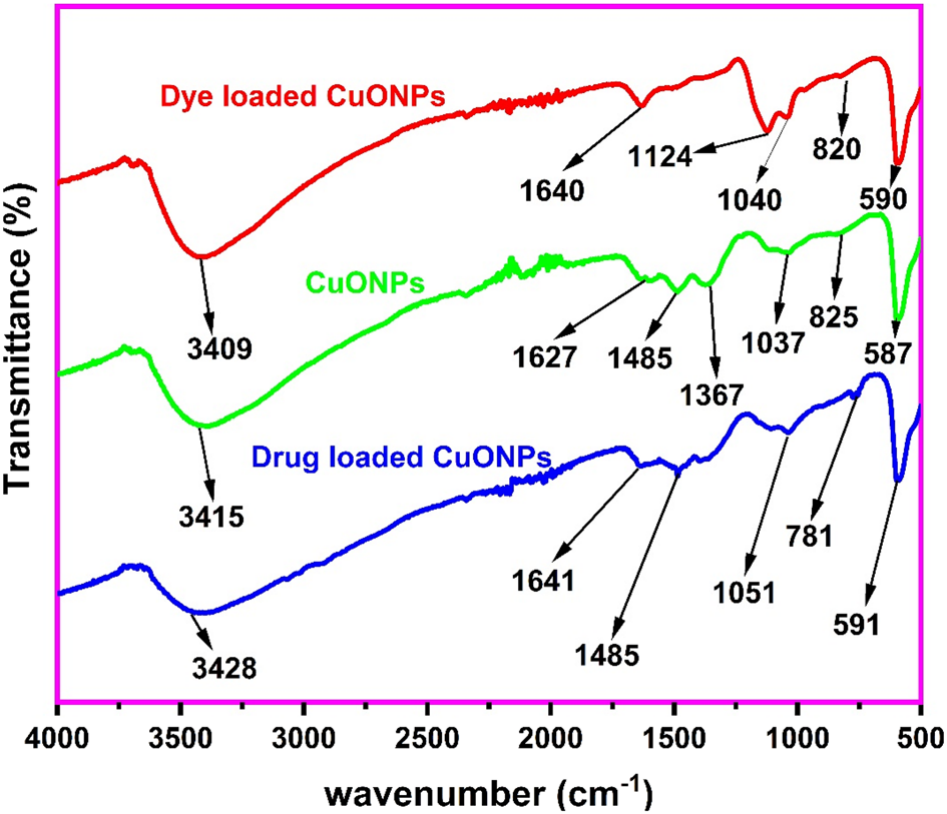
FTIR of copper oxide nanoparticles before and after the uptake of thiazolyl blue and paracetamol.

The biosynthesized CuONPs were characterized by the FTIR to ascertain the surface functional groups which would influence the adsorption of the dye and pharmaceutical as shown in Fig. 1. The broad absorption at 3415 cm^−1^ corresponds to the OH stretching vibration^27^, while absorption at 1627 cm^−1^ is due to the C=C stretching^41^. The moderate absorption bands at 1485 cm^−1^, 1367 cm^−1^ and 1037 cm^−1^ can be ascribed to the C–H bending, OH bending and C–O stretching vibrations, respectively^41,42^, acquired from the leaf extracts of *Platanus occidentalis*, since pure CuONPs do not contain C and H. In addition, the band at 825 cm^−1^ and the intense absorption at 587 cm^−1^ corresponds to the Cu–O stretching vibrations, which confirms the successful synthesis of CuONPs^43,44^. The observed FTIR functionalities of the biosynthesized CuONPs showed that the biological components in the leaf extract were effective in the stabilization and synthesis of the nanoparticles^42^. This is due to the associated presence of the functionals groups of the leave extract with the biosynthesized nanoparticles. After, the adsorption of thiazolyl blue on the CuONPs, shifts in the OH band from 3415 to 3409 cm^−1^, the C=C vibration from 1627 to 1640 cm^−1^, the C–O band from 1037 to 1040 cm^−1^ and the Cu–O stretching from 825 to 820 cm^−1^ and from 587 to 590 cm^−1^ indicate the utilization of these functional groups in the dye uptake. Similarly, for the adsorption of paracetamol, bands shift from 3415 to 3428 cm^−1^, 1627 to 1641 cm^−1^, 1037 to 1051 cm^−1^, 825 to 781 cm^−1^ and 587 to 591 cm^−1^ also indicated the use of the OH, C=C, C–O and Cu–O functionalities for the drug removal. These results showed that the biosynthesized CuONPs contain actives functional groups for the efficient sequestration of the dye and pharmaceutical pollutant from solution.

**Figure 2:**
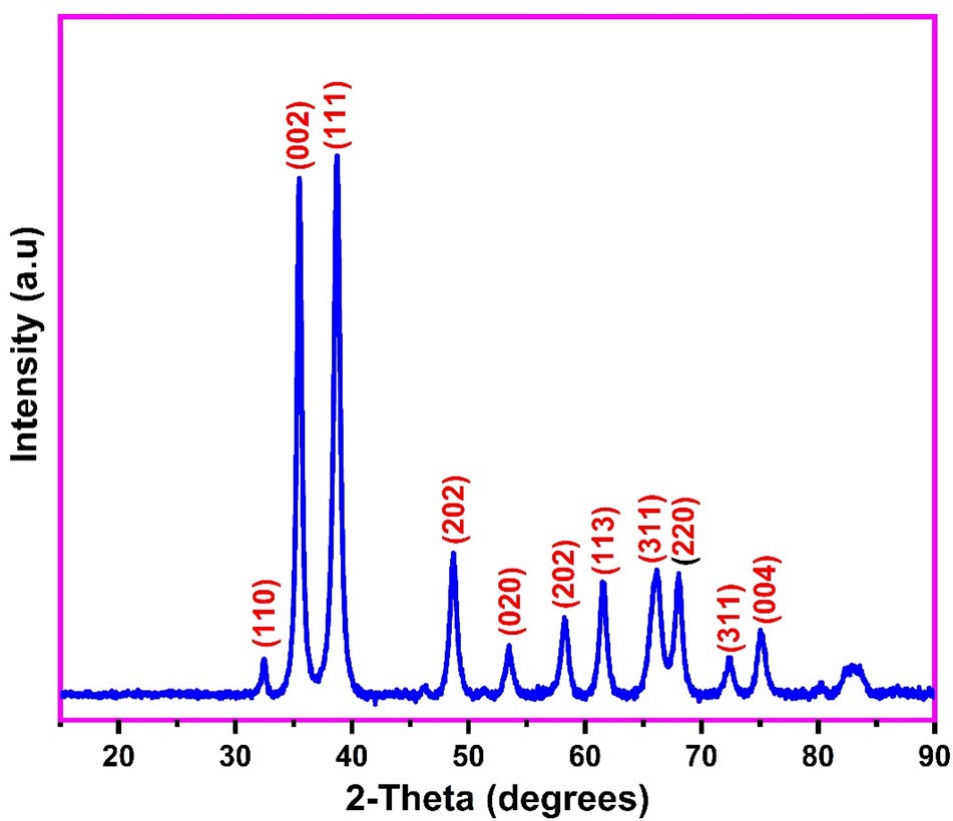
The XRD of the biosynthesized copper oxide nanoparticles.

The XRD spectra of the biosynthesized CuONPs was used to identify the phases and crystalline nature of the adsorbent as shown in Fig. 2. The diffraction peaks at 2θ values of 32.62°, 35.73°, 38.91°, 48.85°, 53.71°, 58.34°, 61.63°, 66.27°, 68.14°, 72.51° and 75.11° correspond to the (110), (002), (111), (202), (020), (202), (113), (311), (220), (311) and (004) monoclinic phases of CuO respectively, as indexed in JCPDS-No-48-1548^45^. The occurrence of these diffractions clearly confirms the successful and efficient green synthesis of the CuONPs using the aqueous leaf extract of *Platanus occidentalis*. The average crystalline size of the biosynthesized CuONPs was calculated from the Debye–Scherrer formular (D = kλ/βcosθ). Where D is the crystalline size, k (0.9) is the Scherrer’s constant, λ (0.1542 nm) is the wavelength of the X-ray used, β corresponds to the full width at half-maximum diffraction peak and θ is the diffraction angle^40^. The average crystalline size of the biosynthesized CuONPs was estimated as 76.4 nm from the intense peak at 2θ of 38.91. The larger crystalline size of our prepared CuONPs in comparison to 13.07 nm^42^, 9.34 nm^46^ and 15 nm^41^ reported is evident from the sharper and more intense peaks of the as-prepared nanoparticles.

**Figure 3:**
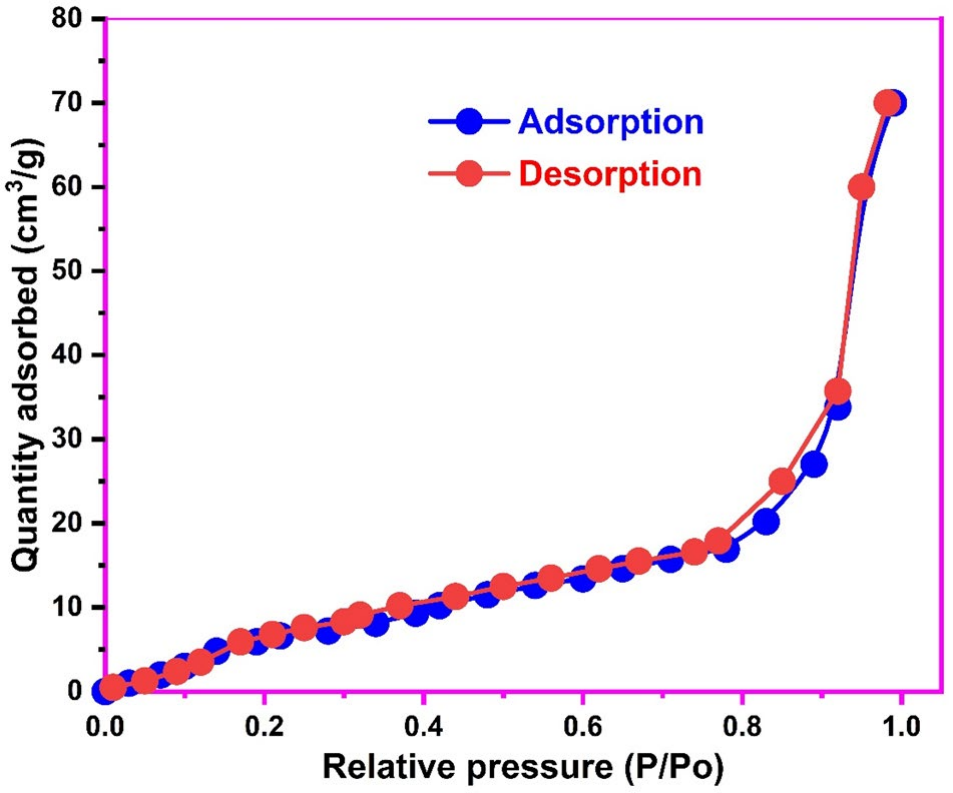
The N_2_ adsorption–desorption isotherm of the biosynthesized copper oxide nanoparticles.

The nitrogen adsorption desorption isotherm at 77 K used to estimate the surface area and textural characteristics of the biosynthesized CuONPs is shown in Fig. 3. As observed, the isotherm corresponds to the type-IV IUPAC classification of porous solids^47^ with a BET surface area of 58.76 m^2^/g. This surface area is close to that (52.11 m^2^/g) reported for the thermal refluxing synthesis^48^ and higher than 42.67 m^2^/g for the thermal synthesis^49^ of CuONPs. Moreover, a pore volume of 0.136 cm^3^/g and pore size of 8.13 nm was obtained from the Barrett-Joyner-Halender method for the biosynthesized CuONPs. Hence, the high surface area and potent textural characteristics of the CuONPs would be beneficial for the efficient uptake of thiazolyl blue and paracetamol from solution.

**Figure 4:**
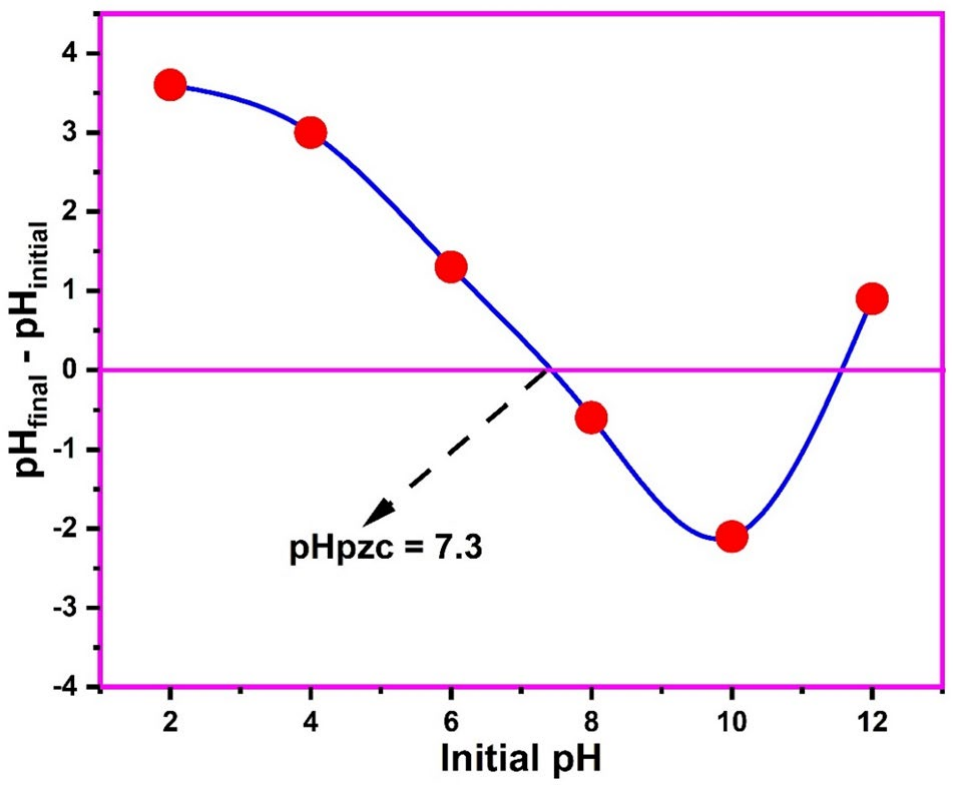
The pH point of zero charge of the biosynthesized copper oxide nanoparticles.

The pH point of zero charge (pHpzc) of the biosynthesized CuONPs as determined by the pH drift method is shown in Fig. 4. The pHpzc is the pH at which the net charge on the surface of the nanoparticles is zero^50^. Usually, at pH values lower than the pHpzc of the nanoparticles, the surface of the material would be positive having strong affinity for negatively charged or anion pollutant species. On the other hand, at pH values higher than the pHpz, the surface becomes negative, thus attracting positively charge or cationic pollutants^13^. The pHpzc of the biosynthesized CuONPs was 7.3 indicating that the nanoparticle surface would be positive at acidic pH values up until pH 7.3 after which it would become negative. Thus, optimum adsorption of the cationic thiazolyl blue from solution onto the CuONPs is likely to be achieved at pH values in the alkaline region. For neutral paracetamol molecules the pKa would play a significant role as it determines the pH of dissociation of the molecules in solution to obtain the charged species.

**Figure 5:**
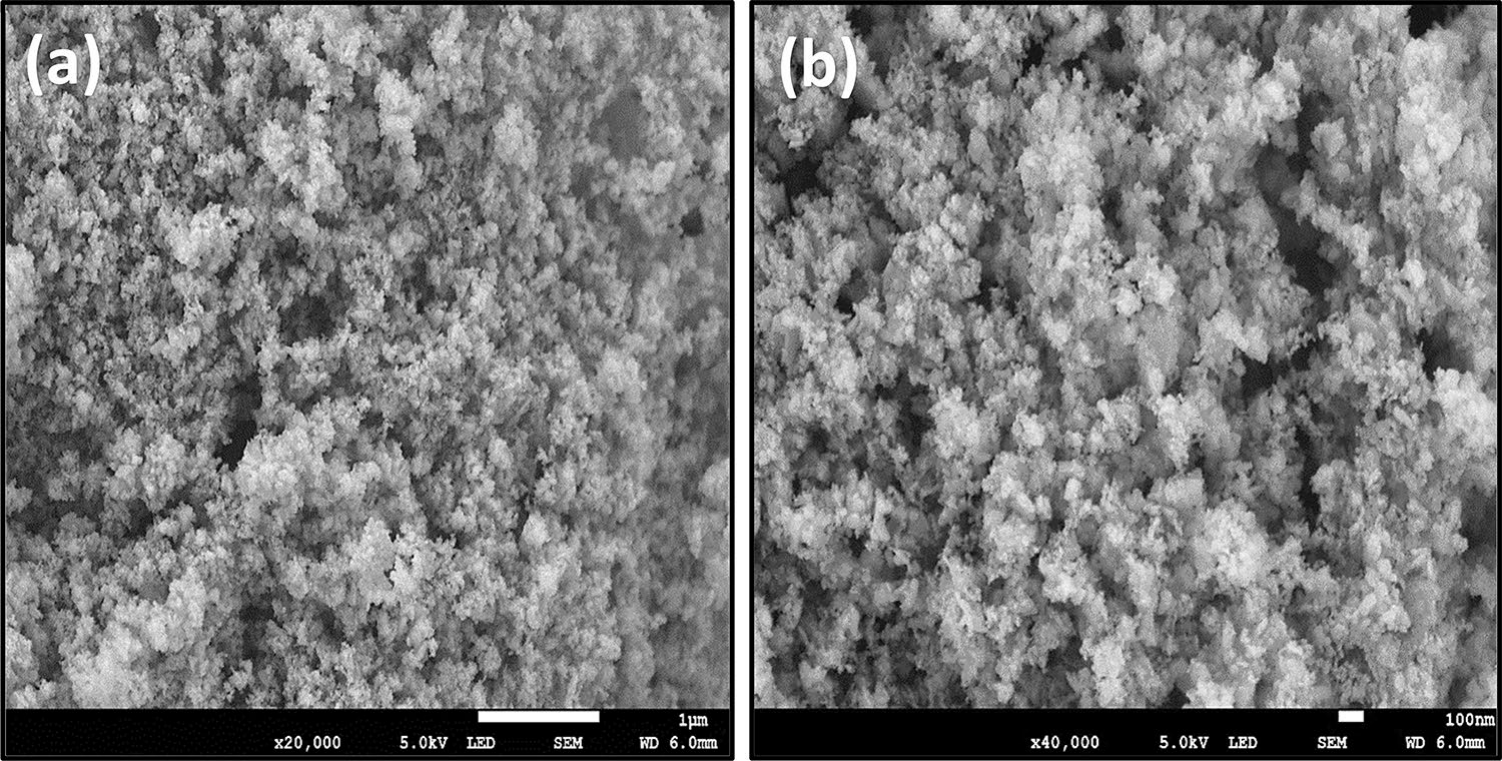
The SEM images of the biosynthesized copper oxide nanoparticles at (**a**) 1 µm and (**b**) 100 nm.

**Figure 6:**
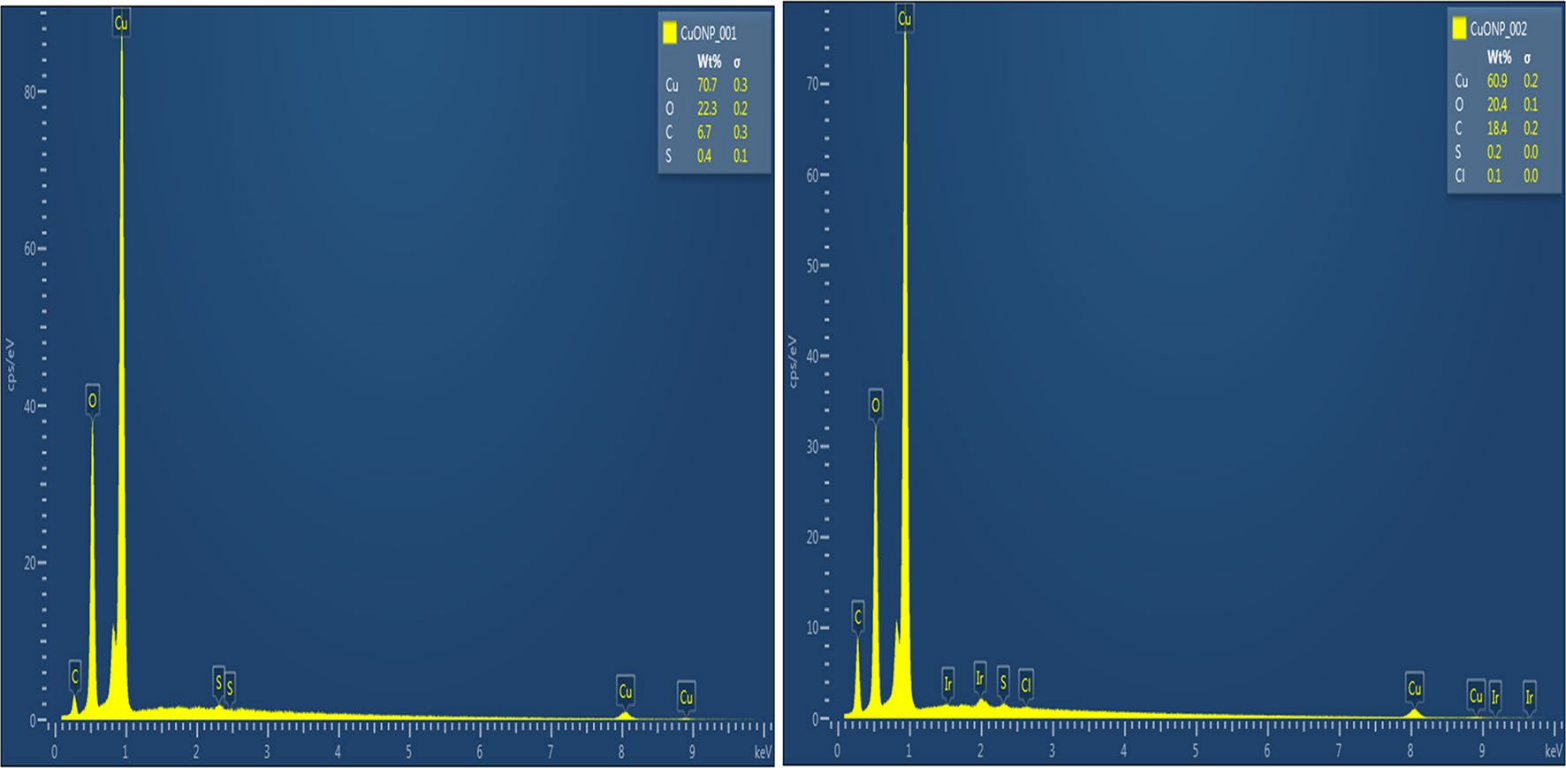
The EDX spectra of the biosynthesized copper oxide nanoparticles.

The SEM was used to examine the surface morphology and porous nature of the nanoparticles. Figure 5 shows the SEM images of the biosynthesized CuONPs at different magnifications. As observed, the biosynthesized nanoparticles exhibited an irregular and crystalline surface morphology containing particles of various sizes and shapes. The average size of the nanoparticles was 82.13 nm, which is close to that obtained from the XRD. This result is similar to that reported for the synthesis of CuONPs using the aqueous leaf extract of *Phyllanthus emblica*^29^. Moreover, the CuONPs had a somewhat porous structure with a slight agglomerated morphology. The use of biological components during the synthetic process may be the cause of the modest agglomeration in the as-produced nanoparticles. The hydroxyl groups of diverse phenolic chemicals in the plant extract engage in intermolecular hydrogen bonding, which lead to agglomeration^51^. This observation is consistent with the report on the biosynthesis of CuONPs using the leaf extract of *Wedelia urticifolia*^27^. In addition, the elemental composition of the biosynthesized CuONPs was accessed from the EDX spectra as shown in Fig. 6. As expected, the most intense peaks were observed for copper and oxygen atoms in the spectra. Moreover, the major constituents of the as-prepared CuONPs are copper, oxygen and carbon with an average composition of 65.8%, 21.4% and 12.6% respectively. This apparently confirms the successful green synthesis of the CuONPs using the aqueous leaf extract of *Platanus occidentalis*. The carbon component of the nano-adsorbent was acquired from the biobased components of the plant extract. This result is consistent with the EDX of CuONPs reported by other researchers which showed the highest composition for copper and oxygen atoms^29,42,49,52^.

### Influence of solution pH

**Figure 7:**
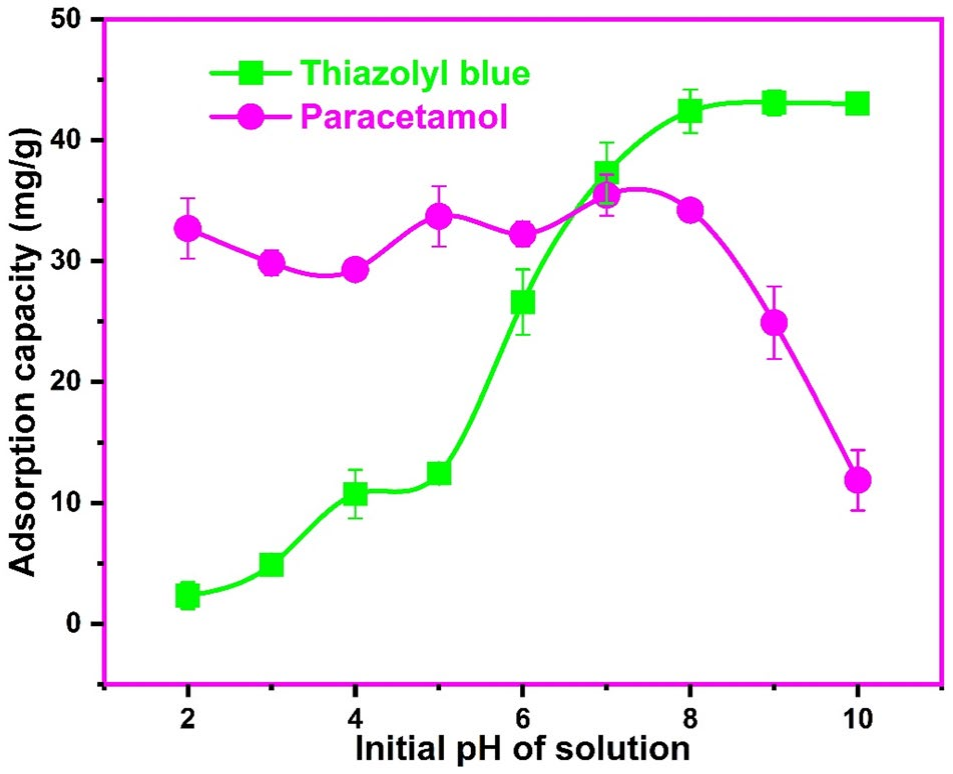
The influence of pH on the uptake of thiazolyl blue and paracetamol on the biosynthesized copper oxide nanoparticles (Sonication time 120 min; temperature 300 K; concentration 100 mg/L).

Due to the fact that the surface charge and speciation of materials and pollutants in solution are affected by the pH, this factor has a substantial impact on the interaction and consequently adsorption. Additionally, the presence of various functional groups on the adsorbate and adsorbent, which are typically protonated/deprotonated with changing pH, could create electrostatic interaction between the two phases^3^. In this regard, the influence of solution pH on the adsorption of thiazolyl blue and paracetamol onto the biosynthesized CuONPs was investigated as shown in Fig. 7. For the adsorption of thiazolyl blue, we observed an increase in the adsorption capacity of CuONPs from 2.35 to 42.4 mg/g with the increase in solution pH from 2.0 to 8.0 after which the uptake was somewhat constant with further increase in pH. It is apparent that higher pH values favored the adsorption of the cationic dye on the nano-adsorbent as envisaged from the pHpzc characterization. Thus, at pH values lower than the pHpzc of 7.3 of CuONPs, the positive nanoparticle surface repelled the cationic thiazolyl blue species causing low removal. As the pH increased, there was an increase in the surface negative charge of CuONPs, which was negative at pH 8.0, enhancing electrostatic attraction of the cationic dye species. Therefore, electrostatic interaction was a major mechanism in the adsorption of thiazolyl blue on the biosynthesized CuONPs. Conversely, for the adsorption of paracetamol, there was no significant change in adsorption from pH 2.0 to 8.0 but a decrease in adsorption was noticed with further increase in pH. This phenomenon could be explained in terms of the pKa value of 9.38 for paracetamol. Thus, the paracetamol exists in its molecular form at pH values below 9.38 and in its dissociated or charge species at higher pH values. Thus at basic pH, there was dissociation of the paracetamol, resulting in an increasing number of it anionic species which repels the negatively charge CuONPs causing lower adsorption^18,53^. Hence, we selected a neutral pH of 7.0 for the adsorption of paracetamol. Some researchers also reported a negligible influence of pH on the adsorption of other safranin-O and neutral red pollutants on CuONPs^54,55^. At the optimum adsorption observed at lower pH values of 2.0 to 8.0, it is apparent that electrostatic interaction did not have much influence on paracetamol adsorption on the biosynthesized CuONPs. Therefore, intermolecular mechanisms like hydrogen bonding and Van der Waals interactions which are unaffected by charged species in the solution probably govern the adsorption of paracetamol^55^.

### Adsorption isotherm modeling

**Figure 8:**
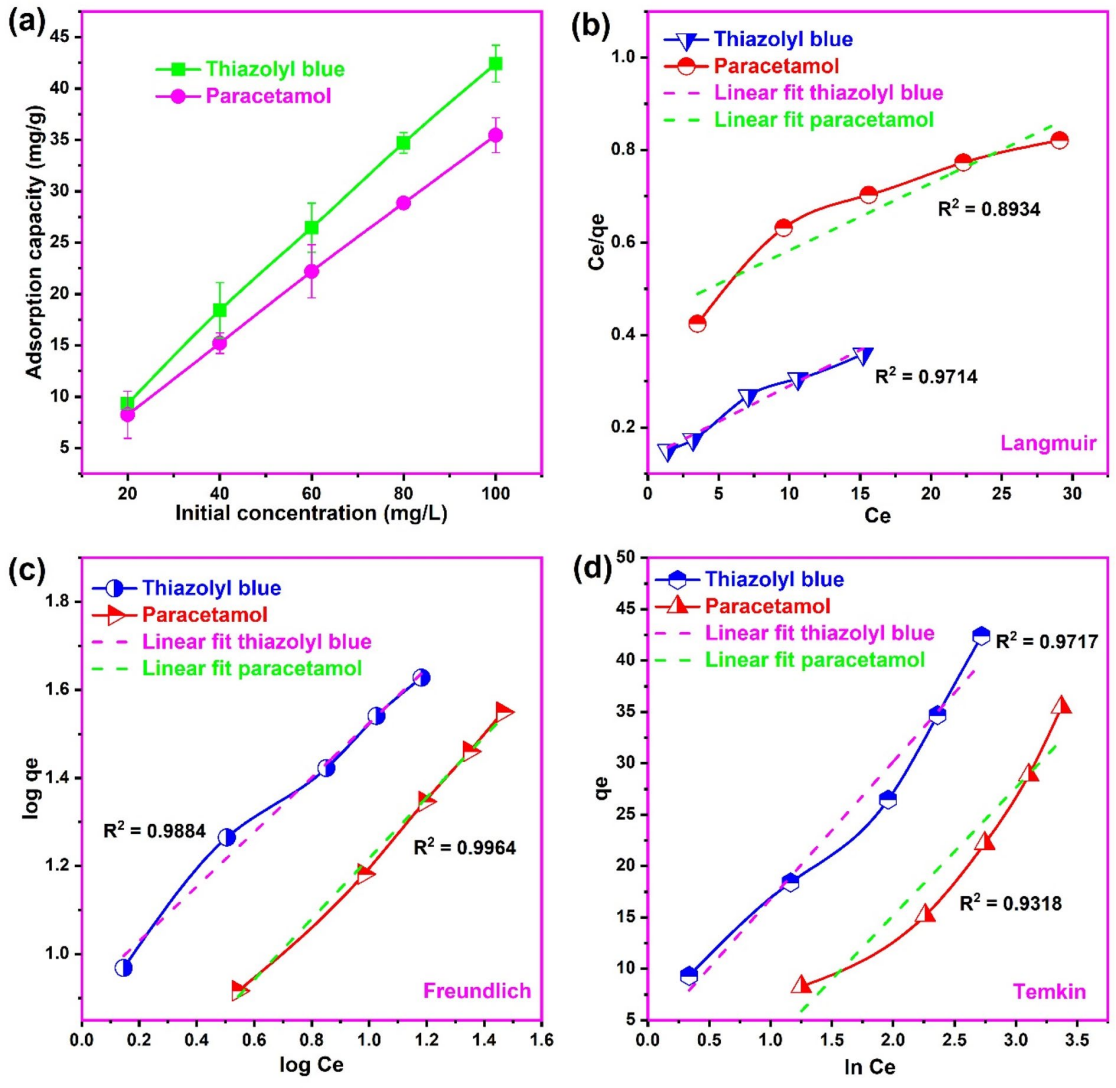
(**a**) The influence of concentration, (**b**) Langmuir (**c**) Freundlich and (**d**) Temkin plots for the uptake of thiazolyl blue and paracetamol on the biosynthesized copper oxide nanoparticles (pH 8.0 for thiazolyl blue and pH 7.0 for paracetamol; Sonication time 120 min; temperature 300 K).

The isotherm modeling of an adsorption process provides vital information on the surface characteristics of the adsorbent, nature of interaction and the affinity between the adsorbate and the adsorbent^56^. In this regard, the isotherm modeling of thiazolyl blue and paracetamol adsorption onto the biosynthesized CuONPs was evaluated as presented in Fig. 8. With increase in the concentration from 20 to 100 mg/L, we observed a continuous increase in the adsorption capacity of CuONPs from 9.3 to 42.4 mg/g and from 8.25 to 35.45 mg/g for the uptake of thiazolyl blue and paracetamol, respectively. Therefore, the concentration of 100 mg/L was utilized in the adsorption experiments in order to ensure maximum use of the active sites of CuONPs. The increase in adsorption capacity with concentration could be attributed to the higher driving force generated by the higher concentration gradient which promotes better interaction with the active sites of the biosynthesized CuONPs^57^. This result is consistent with the report on the adsorption of paracetamol onto polymer-brush grafted mesoporous silica nanoparticles^22^ and the adsorption of thiazolyl blue onto plant leaf derived biochar^14^.

The isotherm analysis of the equilibrium data was modelled by the Langmuir, Freundlich and Temkin isotherms as described in the supplementary document. The adsorption isotherm plots obtained for the adsorption of thiazolyl blue and paracetamol onto the biosynthesized CuONPs are illustrated in Fig. 8. The calculated isotherm parameters obtained are presented in Table 1. The best fit model to the adsorption process correlates with the model with the highest coefficient of determination (R^2^) value and the lowest sum square error (SSE). As observed, for the adsorption of thiazolyl blue, the Freundlich model presented the highest R^2^ of 0.9884, while the Langmuir model provided the lowest SSE of 0.001. However, the SSE of the Freundlich model of 0.002 was still low indicating that this model was more applicable to the adsorption of thiazolyl blue onto CuONPs. For paracetamol adsorption, it is apparent that the Freundlich model was best fitted to the adsorption data based on the lowest SSE and highest R^2^ compared to the Langmuir and Temkin models. Therefore, the best fit presented by the Freundlich isotherm for both pollutants indicate a multilayer adsorption of the dye and pharmaceutical onto a heterogenous CuONPs surface^58^. This heterogenous deduction was corroborated by the SEM images of the biosynthesized nanoparticles which showed an irregular surface morphology with particles of various sizes and shapes. Moreover, the multilayer loading also indicates that physisorption must have played a dominant role in the removal of both pollutants from solution^59^.

**Table 1:** Isotherm parameters for the uptake of thiazolyl blue and paracetamol onto copper oxide nanoparticles.

Model	Parameter	Thiazolyl blue	Paracetamol
Langmuir	K_L_ (L/mg)	0.102	0.035
	q_L_ (mg/g)	72.46	64.52
	R^2^	0.9714	0.8934
	SSE	0.001	0.010
Freundlich	N	1.615	1.450
	K_F_ (L/g)	8.042	3.373
	R^2^	0.9884	0.9964
	SSE	0.002	0.001
Temkin	A (L/g)	1.287	0.458
	B (mg/g)	13.39	12.45
	R^2^	0.9719	0.9318
	SSE	19.14	31.61

The affinity between the pollutants in solution and the biosynthesized CuONPs can be evaluated from the Freundlich n value. Generally, n values between 1 and 10 corresponds to a favorable adsorption process^32^, which indicates good interaction between the adsorbate and adsorbent. As observed the Freundlich n value for both paracetamol and thiazolyl blue were in the favorable range, indicating the potentials of the biosynthesized CuONPs as an efficient material for the decontamination of water polluted with these substances. In addition, the Langmuir separation factor [R_L_ = 1/(1 + K_L_C_o_)] also provides insight into the affinity between the adsorbent and adsorbate. Where K_L_ and C_o_ are the Langmuir constant and initial concentration of the adsorbate respectively. Usually, R_L_ between 0 and 1 is attributed to a favorable adsorption while R_L_ > 1 corresponds to an unfavorable one^18^. For the adsorption of thiazolyl blue and paracetamol on CuONPs, the R_L_ values ranging from 0.089 to 0.329 and from 0.222 to 0.588 were obtained respectively, which corroborates the favorable adsorption of both substances on the nano-adsorbent.

The maximum monolayer adsorption capacities of 72.46 mg/g and 64.52 mg/g were obtained for the uptake of thiazolyl blue and paracetamol on CuONPs, respectively, indicating the higher adsorption of the former. This could be due to the stronger electrostatic interaction mechanisms involved in the uptake of the dye species in comparison to the weaker H-bonding and Van der Waals forces predominant in paracetamol adsorption. Moreover, the adsorption of paracetamol on CuONPs is higher than that obtained for other efficient adsorbents like sugar cane bagasse (0.121 mg/g)^60^, metal–organic-framework-MIL type (1.87 mg/g)^61^, activated carbon (43.5 mg/g)^62^ and acid modified clay (43.92 mg/g)^20^. Similarly, the uptake of thiazolyl blue on the CuONPs was higher than 48.80 mg/g reported for American sycamore derived biochar^14^. These findings support the potentials of the biosynthesized CuONPs for environmental remediation of these substances from contaminated water.

### Kinetics of adsorption

**Figure 9:**
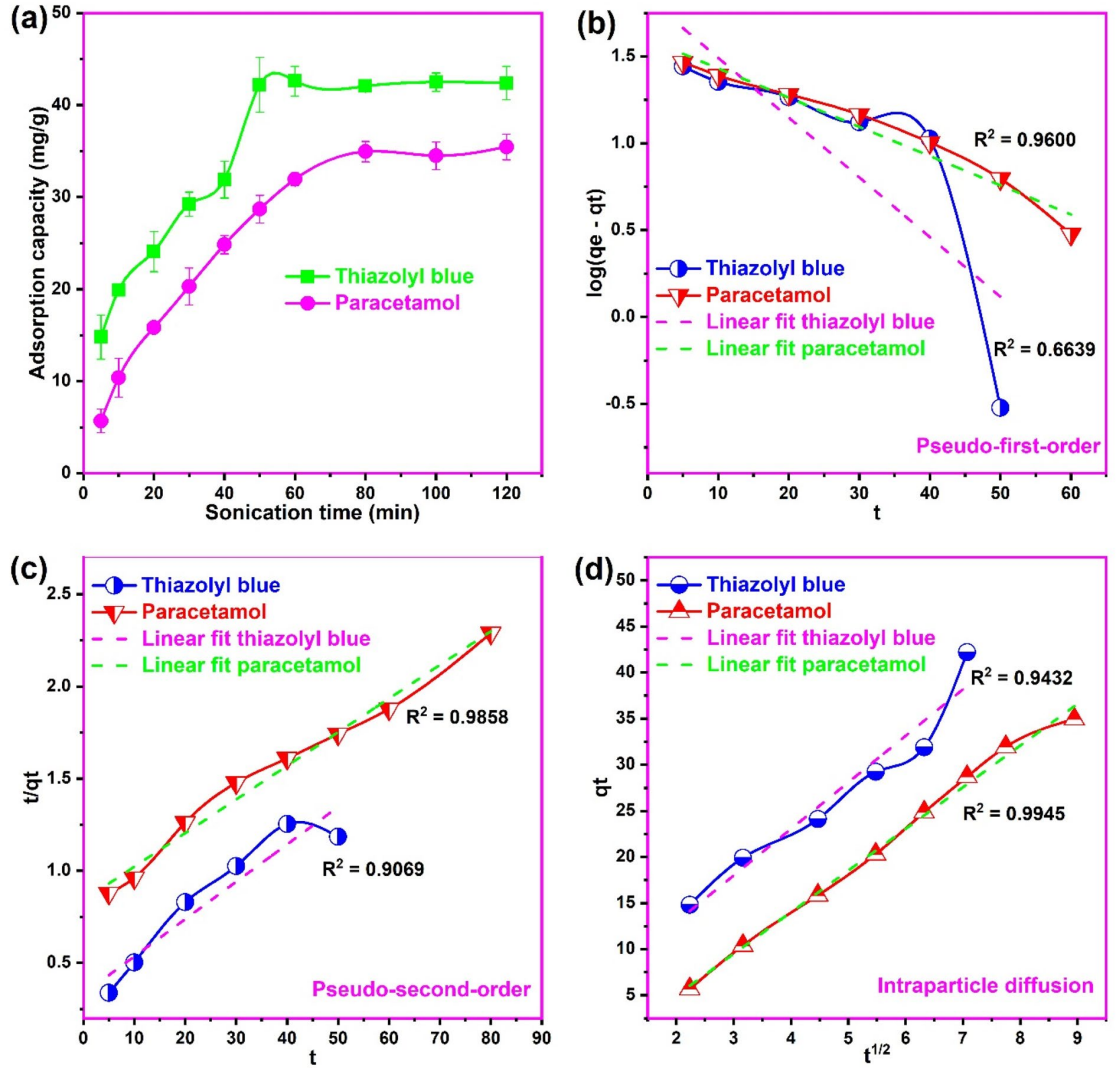
(**a**) The influence of sonication time, (**b**) Pseudo-first-order (**c**) Pseudo-second-order and (**d**) Intraparticle diffusion plots for the uptake of thiazolyl blue and paracetamol on the biosynthesized copper oxide nanoparticles (pH 8.0 for thiazolyl blue and pH 7.0 for paracetamol; concentration 100 mg/L; temperature 300 K).

The kinetics analysis of an adsorption process provides vital information on the rate of removal and mechanism useful in the design of adsorption systems^63^. The kinetic studies on the adsorption of thiazolyl blue and paracetamol onto the biosynthesized CuONPs is shown in Fig. 9. We observed an increase in the adsorption of thiazolyl blue from 14.8 to 42.2 mg/g with an increase in sonication time from 5 to 50 min. Likewise, an increase in sonication time from 5 to 80 min resulted to an increase in the adsorption of paracetamol from 5.7 to 34.9 mg/g. Further increase in time beyond 50 min for thiazolyl blue and 80 min for paracetamol resulted in a negligible change in the adsorption of both pollutants on CuONPs. The trend could be explained in terms of the availability of vacant sites on the nanoparticles and the saturation of the active sites^64^. At the initial stages of adsorption, there were abundant active sites on the biosynthesized CuONPs causing rapid adsorption of the pollutants from solution. However, at equilibrium the active sites were saturated with the dye and pharmaceutical molecules leading to no further removal. This result is consistent with the findings on the adsorption of safranin-O^54^ and methyl orange^43^ on green synthesized CuONPs. The sonication time of 120 min was chosen to enable equilibrium adsorption of both pollutants from solution. Moreover, the kinetic result indicated a faster uptake of the dye molecules on CuONPs when compared to the adsorption of paracetamol. Therefore, after the initial rapid adsorption on the vacant active sites of CuONPs, the bulkier thiazolyl blue molecules encounter greater resistance in the intraparticle competition which hindered further removal. On the other hands, the smaller paracetamol molecules had less intraparticle diffusion resistance causing further removal with time until the active sites were completely saturated^65^. As a result, intraparticle diffusion is likely the rate controlling mechanism in the adsorption of paracetamol.

The kinetic modelling of adsorption of the dye and pharmaceutical on the biosynthesized CuONPs was evaluated by the Pseudo-first-order (PFO), Pseudo-second-order (PFO) and intraparticle diffusion (ID) model equations as presented in the supplementary document. The obtained kinetic model plots and parameters are presented in Fig. 9 and Table 2, respectively. For the adsorption of thiazolyl blue, it is apparent that the process was well correlated to the PSO model based on the higher R^2^, the lower SSE and the closer the model qe to the experimental one, compared to the PFO model. For the adsorption of paracetamol, both the PFO and PSO models presented good fits to the kinetic data. Although the model qe value of the PFO model was closer the experimental value, the higher R^2^ and lower SSE of the PSO model indicated a greater conformity of the adsorption to the PSO model. The best fit of the PSO model to the process corroborates the findings of most researchers on the uptake of various pollutants on CuONPs^27,29,32,54,55,58,65–67^. Therefore, based on its premise, the number of active sites occupied in the adsorbent is proportional how much adsorbate would be absorbed^54,55,68^.

**Table 2:** Kinetic parameters for the uptake of thiazolyl blue and paracetamol onto copper oxide nanoparticles.

Model	Parameter	Thiazolyl blue	Paracetamol
	qe_exp_ (mg/g)	42.50	34.95
Pseudo-first-order	K_1_ (min^−1^)	0.079	0.039
	qe_cal_ (mg/g)	68.57	39.76
	R^2^	0.6639	0.9600
	SSE	0.911	0.029
Pseudo-second-order	K_2_ (mg/g min)	6.1 × 10^−2^	3.9 × 10^−4^
	qe_cal_ (mg/g)	49.31	54.82
	R^2^	0.9069	0.9858
	SSE	0.064	0.023
Intraparticle diffusion	K_d_ (mg/g min^−1/2^)	5.049	4.519
	C	2.835	4.079
	R^2^	0.9432	0.9945
	SSE	1.047	0.018

The PSF and PSO does not provide information on the mechanism of diffusion, therefore, the kinetic diffusion mechanism of the uptake on the biosynthesized CuONPs was evaluated by the ID model^54^. It is evident from the high R^2^ obtained for both pollutants that intraparticle diffusion was involved in the adsorption onto CuONPs. However, for the adsorption of paracetamol, the ID model presented the highest R^2^ and lowest SSE compared to the PFO and PSO. This indicates that the ID is the rate controlling mechanism in the adsorption of paracetamol on CuONPs which corroborates our findings on the effect of sonication time on adsorption. However, the occurrence of the intercept (C = 4.079) indicated that the plot did not pass through the origin and that ID is not the only rate controlling mechanism but involves to some extent the boundary layer diffusion^33,69^. This finding is consistent with the result on the adsorption of imidacloprid insecticide on activated carbon loaded with magnetite and CuO nanoparticles^70^.

### Adsorption thermodynamics

**Figure 10:**
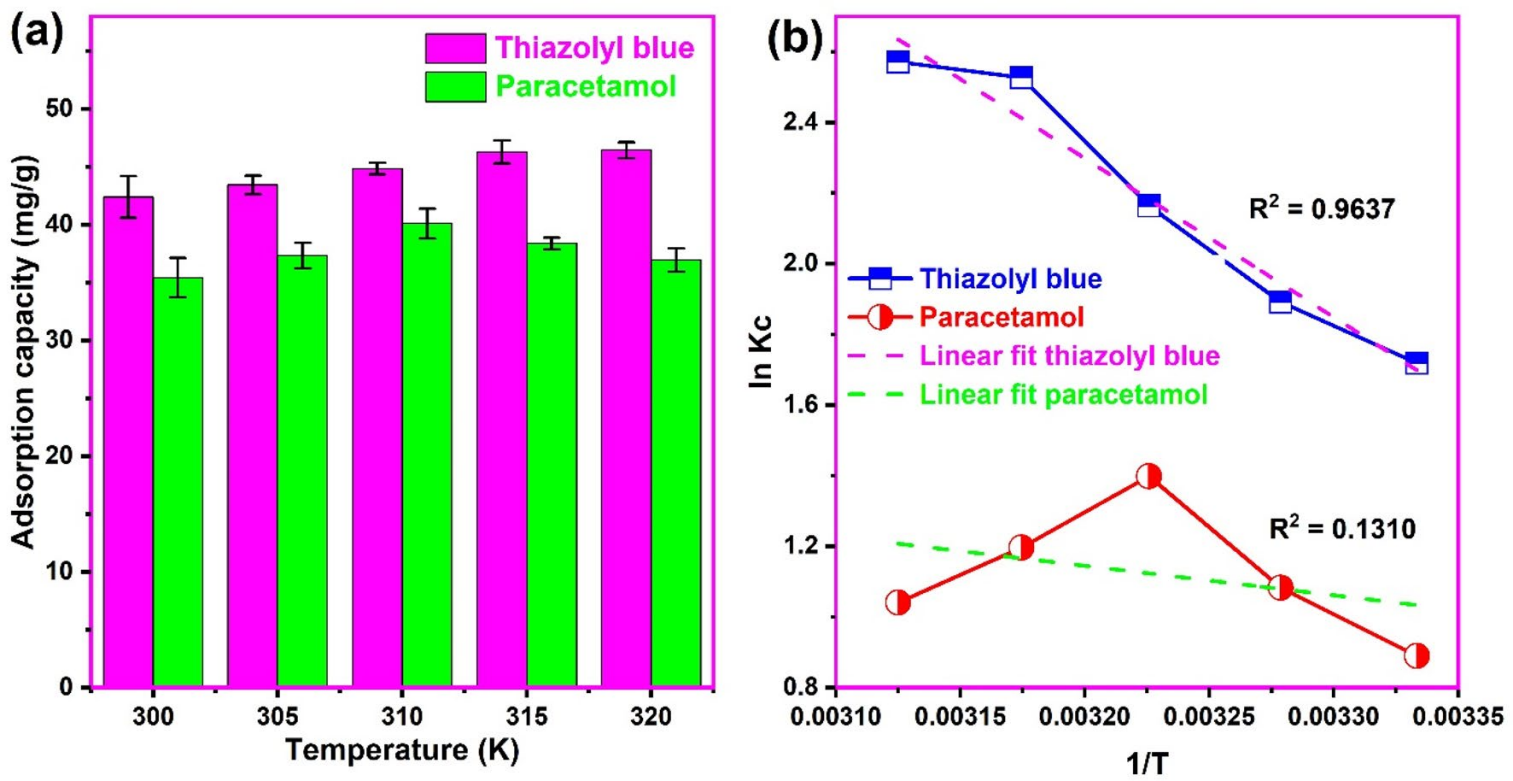
(**a**) The influence of temperature and (**b**) Van’t Hoff’s plot for the uptake of thiazolyl blue and paracetamol on the biosynthesized copper oxide nanoparticles (pH 8.0 for thiazolyl blue and pH 7.0 for paracetamol; concentration 100 mg/L; sonication time 120 min).

The thermodynamic analysis of adsorption provides useful information on the spontaneity, degree of randomness, exothermic/endothermic nature as well as the physical or chemical nature of the adsorption^3^. The thermodynamic evaluation of adsorption of thiazolyl blue and paracetamol onto the biosynthesized CuONPs is shown in Fig. 10. We observed an increase in the adsorption of thiazolyl blue from 42.4 to 46.5 mg/g with an increase in the solution temperature from 300 to 320 K. The increase in adsorption with temperature may be attributed to the dye molecules' rising kinetic energy, which helps them overcome mass transfer barrier and interact more effectively with CuONPs active sites^71^. Additionally, as the temperature rises, the adsorbent's internal structure could be enlarged, allowing more dye molecules to pass through^72^. This result is consistent with the report on the adsorption of acid red 57^49^ and methyl orange^72^ dyes on CuONPs. It is apparent that the uptake of the dye molecule on CuONPs is an endothermic process. In the case of paracetamol, there was an initial increase in adsorption from 35.5 to 40.1 mg/g and then a decrease from 40.1 to 36.9 mg/g with the increase in temperature from 300 to 310 K and from 310 to 320 K, respectively. A similar trend was observed in the adsorption of paracetamol on activated carbon^18^ and the adsorption of Cr(VI) onto magnetic sulfacetamide ethylacetoacetate hydrazone-chitosan Schiff base adsorbent^73^. The subsequent decrease in paracetamol adsorption at higher temperature beyond 310 K could be ascribed to the increasing kinetic energy causing desorption of physically adsorbed molecules from the surface of the nanoparticle^74^. It could also be attributed to the increase in Brownian motion and thermal energy of the pollutant in the bulk solution^73^. Moreover, higher temperature can result in the increasing solubility of paracetamol in solution favoring its interaction with the liquid phase rather than the solid phase nanoparticles^75^.

The thermodynamic parameters such as the Gibbs free energy change (ΔG°), enthalpy change (ΔH°) and entropy change (ΔS°) for the uptake of both pollutants on the biosynthesized CuONPs were calculated from the Gibbs free energy and Van’t Hoffs equations and presented in Table 3. A spontaneous adsorption of thiazolyl blue and paracetamol on the nano-adsorbent was obtained at all temperatures as indicated by the negative ΔG° values.

**Table 3:** Thermodynamic parameters for the uptake of thiazolyl blue and paracetamol onto copper oxide nanoparticles.

Sorbent	T(K)	ΔG° (kJ/mol)	ΔH° (kJ/mol)	ΔS° (J/mol K)	R^2^
Thiazolyl blue	300	− 4.288	37.38	138.7	0.9637
	305	− 4.798			
	310	− 5.578			
	315	− 6.617			
	320	− 6.841			
Paracetamol	300	− 2.221	6.933	31.71	0.1310
	305	− 2.745			
	310	− 3.605			
	315	− 3.135			
	320	− 2.769			

This indicates a feasible use of the biosynthesized CuONPs for the decontamination of water containing these pollutants. This is consistent with the findings on the adsorption of paracetamol on clay^20^ and thiazolyl blue onto biochar^14^. Besides, an increase in randomness at the dye/CuONPs and pharmaceutical/CuONPs interface during the process was indicated by the positive ΔS° of 138.7 J/molK and 31.71 J/molK, respectively. Moreover, the overall adsorption of thiazolyl blue and paracetamol on the biosynthesized CuONPs was endothermic as deduced from their positive ΔH° values irrespective of the slight decrease in adsorption with further increase in temperature above 310 K, observed for the latter. A similar endothermic adsorption of lead (II) ions onto CuONPs was reported^76^. Additionally, the magnitude of ΔH° helps in the classification of the process as physisorption or chemisorption. The ΔH° values less than 40 kJ/mol is said to be dominated by physical adsorption while values higher than 40 kJ/mol can be as ascribed to chemisorption^77^. The ΔH° values obtained indicated that the adsorption of thiazolyl blue and paracetamol on the biosynthesized CuONPs conforms to physical adsorption, which supports the deduction of the isotherm model analysis. This physical adsorption would be beneficial for easy desorption of the pollutants from the loaded nanoparticle. Moreover, the ΔH° of 37.38 kJ/mol obtained for thiazolyl blue was much higher than 6.933 kJ/mol for paracetamol. This was probably due to the predominance of stronger electrostatic interactions in the dye adsorption deduced from the pH study when compared to the weaker intermolecular interaction between paracetamol and the biosynthesized CuONPs.

### Desorption and reusability

**Figure 11:**
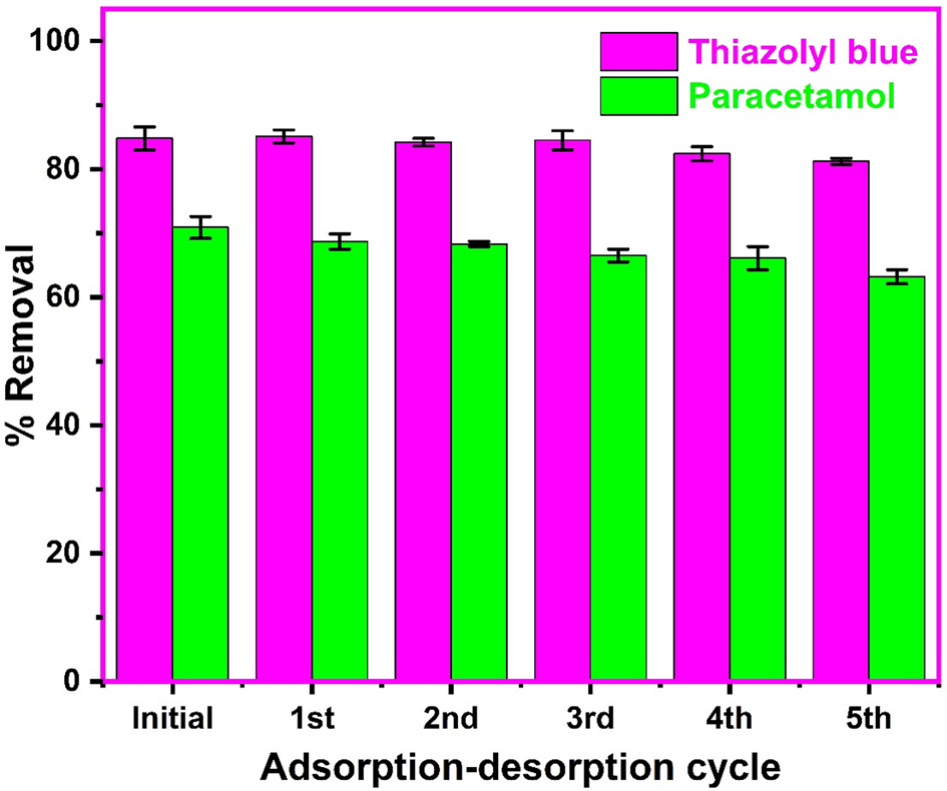
The regeneration and reuse of the biosynthesized copper oxide nanoparticles for the uptake of thiazolyl blue and paracetamol (pH 8.0 for thiazolyl blue and pH 7.0 for paracetamol; concentration 100 mg/L; temperature 300 K, sonication time 120 min).

For the best applicability, sustainability, and commercialization, a viable sorbent should exhibit effective dye adsorption behavior in addition to good regeneration and reuse^78^. Additionally, the eluting solvent employed in the regeneration process needs to be inexpensive and non-harmful to the adsorbent. Sodium hydroxide and hydrochloric acid solution have been reported as suitable eluents for the desorption of paracetamol^53^ and thiazolyl blue^14^, respectively. These eluents were utilized for the desorption of the pollutants from the loaded biosynthesized CuONPs. Figure 11 illustrates the reusability of the CuONPs for the adsorption of the dye and pharmaceutical after five adsorption–desorption. We observed a slight loss in adsorption of thiazolyl blue from 84.1 to 81.2% and from 70.9 to 63.2% for paracetamol in the initial uptake to the fifth cycle of reuse. The result indicated that the biosynthesized CuONPs were effectively regenerated and reused for the uptake of both pollutants from solution. This finding is consistent with the reports on the successful reuse of CuONPs for the adsorption of other pollutants from solution^48,49,54,55^.

## Conclusions

The adsorption of thiazolyl blue and paracetamol from solution onto *Platanus occidentalis* mediated green synthesized copper oxide nanoparticles (CuONPs) was studied. The characterization showed the successful synthesis of CuONPs using the aqueous extract of *Platanus occidentalis.* The pH of solution had a significant influence on the adsorption of thiazolyl blue unlike the adsorption of paracetamol. Variations in the adsorption of both pollutants on CuONPs were observed with changes in concentration, sonication time and solution temperature. The adsorption of both pollutants was well fitted with the Freundlich and Pseudo-second-order models and involved intraparticle diffusion mechanism. Adsorption thermodynamics showed an endothermic, random, spontaneous and physical adsorption of both pollutants on CuONPs. The loaded CuONPs were successfully regenerated and reused. Moreover, the biosynthesized CuONPs exhibited higher adsorption capacity for thiazolyl blue and paracetamol than many efficient adsorbents which indicated it potentials as a viable nano-adsorbent for water purification.

## Supplementary Information

Supplementary Information.

## Data Availability

The datasets used and/or analyzed during the current study are available from the corresponding author on reasonable request.
