# Supplementary material for: Efficient adsorptive removal of paracetamol and thiazolyl blue from polluted water onto biosynthesized copper oxide nanoparticles
Source: Sci Rep. 2023 Jan 17;13:859. doi: 10.1038/s41598-023-28122-0 (PMC9845337; doi:10.1038/s41598-023-28122-0)
Supplement: Supplementary file 1 — Supplementary Information. [file 41598_2023_28122_MOESM1_ESM.docx]

**Efficient adsorptive removal of paracetamol and thiazolyl blue from polluted water onto biosynthesized copper oxide nanoparticles**

**Kovo G. Akpomie^1,2^, Jeanet Conradie^1^**

^1^Physical Chemistry Unit, Department of Chemistry, University of the Free State, Bloemfontein, South Africa.

^2^Industrial/Physical Chemistry Unit, Department of Pure & Industrial Chemistry, University of Nigeria, Nsukka, Nigeria.

E-mail: [kovo.akpomie@unn.edu.ng](mailto:kovo.akpomie@unn.edu.ng)

**SUPPORTING INFORMATION**

**Percentage removal and adsorption capacity**

The percentage removal of thiazolyl blue and paracetamol and the adsorption capacity (mg/g) of the CuONPs were calculated by the respective equations ^1^:

$$\%Removal =\left( \frac{\left( C_{o}-C_{e} \right)}{C_{o}} \right)*100 (1)$$

$$qe=\frac{\left( C_{o}-C_{e} \right)V}{m} (2)$$

Where *C_o_* and *C_e_* in mg/L represent the initial and residual concentrations of thiazolyl blue or paracetamol in solution, *m* (g) is the mass of the CuONPs used, and *V* (L) is the volume of solution.

**Isotherm modelling**

The affinity between thiazolyl blue or paracetamol and the synthesised CuONPs was analysed by the application of the Langmuir, Freundlich, and Temkin isotherms ^2,3^. The Freundlich isotherm related to a heterogeneous multilayer abstraction is given as:

$$\log q_{e}= \log K_{F}+ \left( \frac{1}{n} \right)\log C_{e} (3)$$

The Langmuir model which represents homogenous monolayer adsorption is expressed as:

$$\frac{C_{e}}{q_{e}}=\frac{1}{q_{L}K_{L}}+\frac{C_{e}}{q_{L}} (4)$$

The Temkin isotherm assumes the surface coverage of adsorbent is dependent on the removal

energy and is given as:

$$q_{e}=B\ln A+B\ln C_{e} (5)$$

Where *n* and *K_F_* (L/g) denote the Freundlich adsorption intensity and capacity respectively, *C_e_* (mg/L) is the adsorbate concentration at equilibrium, *K_L_* (L/mg) is the Langmuir constant and *q_L_* (mg/g) corresponds to the maximum monolayer uptake. The constants *A* (L/mg) and *B* correspond to Temkin’s adsorption binding energy and heat energy respectively.

**Kinetic Modelling**

The kinetics of thiazolyl blue and paracetamol removal onto CuONPs was analysed by the pseudo-first-order (PFO), pseudo second order (PSO), and intraparticle diffusion (ID) equations ^4^. The PFO equation is expressed as:

$$\log\left( q_{e}-q_{t} \right)=\log q_{e}-\frac{K_{1}}{2.303}t (6)$$

The PSO kinetic equation is given as:

$$\frac{t}{q_{t}}=\frac{1}{K_{2}{q_{e}}^{2}}+\frac{t}{q_{e}} (7)$$

The ID also applied to provide information on adsorption diffusion mechanism is expressed as:

$$q_{t}=K_{d}t^{\frac{1}{2}}+C (8)$$

Where *t* (min) is the time of adsorption, *q_t_* (mg/g) is the uptake capacity at a particular time. The constants *K_I_* (min^-1^), *K_2_* (g/mg min), and *K_d_* (mg/g min^1/2)^ represent the PFO, PSO, and ID rate constants, respectively, while *C* is the intercept of the ID model, which shows the deviation of the plot from the origin.

**Thermodynamic analysis**

Adsorption thermodynamics analysis was performed to evaluate the enthalpy change (ΔH^o^), entropy change (ΔS^o^) and Gibb’s free energy change (ΔG^o^) by the application of the given equations ^3^:

$$\Delta G^{0}= -RT \ln K_{c} (9)$$

$$\ln K_{C}=-\left( \frac{\Delta H^{0}}{RT} \right)+\left( \frac{\Delta S^{0}}{R} \right) (10)$$

Where *R* (J/mol K), *T* (K), and *K_c_* correspond to the ideal gas constant, absolute temperature, and distribution coefficient respectively.

**References**

1. Zubair, M. *et al.* Adsorption Behavior and Mechanism of Methylene Blue, Crystal Violet, Eriochrome Black T, and Methyl Orange Dyes onto Biochar-Derived Date Palm Fronds Waste Produced at Different Pyrolysis Conditions. *Water, Air, Soil Pollut.* **231**, 240 (2020).

2. Dawodu, M. O. & Akpomie, K. G. Evaluating the potential of a Nigerian soil as an adsorbent for tartrazine dye: Isotherm, kinetic and thermodynamic studies. *Alexandria Eng. J.* **55**, 3211–3218 (2016).

3. Ezekoye, O. M. *et al.* Biosorptive interaction of alkaline modified Dialium guineense seed powders with ciprofloxacin in contaminated solution: central composite, kinetics, isotherm, thermodynamics, and desorption. *Int. J. Phytoremediation* **22**, 1028–1037 (2020).

4. Akpomie, K. G. *et al.* Attenuation of methylene blue from aqua-media on acid activated montmorillonite of Nigerian origin. *J. Environ. Sci. Manag.* **20**, 17–22 (2017).
